# Predictors for grade 6 reading in children at familial risk of dyslexia

**DOI:** 10.1007/s11881-018-0162-1

**Published:** 2018-07-11

**Authors:** Ellie R. H. van Setten, Britt E. Hakvoort, Aryan van der Leij, Natasha M. Maurits, Ben A. M. Maassen

**Affiliations:** 1 Center for Language and Cognition Groningen (CLCG), Faculty of Arts, University of Groningen, Oude Kijk in ‘t Jatstraat 26, 9712 EK Groningen, the Netherlands; 2 Research School of Behavioural and Cognitive Neurosciences (BCN), University Medical Center Groningen, University of Groningen, Groningen, the Netherlands; 3 Research Institute of Child Development and Education, University of Amsterdam, Amsterdam, the Netherlands; 4 Department of Neurology, University Medical Center Groningen, University of Groningen, Groningen, the Netherlands

**Keywords:** Advanced reading skills, Dyslexia, Familial risk, Reading comprehension, Reading fluency

## Abstract

The present study investigates whether grade 6 reading outcomes, reading fluency, and reading comprehension can be predicted by grade 3 reading fluency, familial risk of dyslexia (FR), and grade 3 reading related skills: rapid automatized naming (RAN), phonological awareness (PA), and vocabulary. In a sample of 150 children, of whom 83 had a parent with dyslexia, correlation and regression analyses were performed. FR, measured on a continuous scale, was by itself related to all outcomes. However, FR did not explain any variance on top of grade 3 reading fluency. Grade 3 reading fluency strongly predicted grade 6 reading fluency and was also related to reading comprehension. RAN improved the prediction of grade 6 reading fluency, though the additional explained variance was small. Vocabulary and PA fully explained the variance that grade 3 reading fluency explained in grade 6 reading comprehension. Vocabulary explained a substantial amount of variance in grade 6 reading comprehension making it an interesting clinical target. As we used continuous measures of reading fluency and FR, our findings are not biased by distinct diagnostic criteria.

Dyslexia is a problem with reading and spelling at the word level that affects about 3–10% of the general population, depending on the diagnostic criteria used (Miles, 2004; Shaywitz, Fletcher, Holahan, & Shaywitz, 1992a; Shaywitz, Shaywitz, Fletcher, & Escobar, 1990; Siegel, 2006). The orthographic transparency of a language influences how dyslexia manifests itself; whereas reading speed is the main concern in transparent languages like Dutch, accuracy difficulties remain longer during reading acquisition in opaque languages like English (Serrano & Defior, 2008; Wimmer, 1993; Ziegler & Goswami, 2005). Although dyslexia is defined as a specific learning disability at the word level, reading comprehension deficits are also more common among children with dyslexia compared to the general population (e.g., Ferrer et al., 2015; Shaywitz et al., 1999). Dyslexia has a partially genetic origin; the incidence of dyslexia is higher in identical twins, compared to fraternal twins (DeFries & Alarcón, 1996). The prevalence of dyslexia in children with a dyslexic parent is also much higher than in the general population, although the shared environment plays a role here, as well. A recent meta-analysis of longitudinal studies revealed that the prevalence of dyslexia in samples of children with a familial risk of dyslexia (FR) is on average 45% (Snowling & Melby-Lervåg, 2016).

The present study involves children from the Dutch Dyslexia Program (DDP), a longitudinal study in which Dutch children with a high FR and a control group with a low FR have been followed from birth (see e.g., van Bergen, de Jong, Plakas, Maassen, & van der Leij, 2012; van der Leij et al., 2013). Early DDP studies focused on FR and the main pre-literacy verbal and nonverbal factors determining reading acquisition and reading difficulties, which is of high clinical relevance for early detection, diagnosis, and intervention. The present study focuses on advanced reading. The study aims to further trace the effect of FR and the main developmental factors involved in determining reading fluency and reading comprehension at the end of primary school. Also for this part of the developmental trajectory, the clinical relevance is high. Prognosis of reading development among children with and without FR is a precondition for effective and efficient allocation of resources. The first important predictor in our study is FR, being the earliest risk factor that can be obtained in a child’s personal history. The question is to what extent FR is still predictive of reading level in grade 6, once reading fluency and reading difficulties can be assessed and diagnosed in grade 3. Thus, we investigated the predictive value of grade 3 reading fluency for the grade 6 outcomes relative to FR, to determine whether the familial effect has already manifested itself by grade 3, or the reading development between grade 3 and 6 is still influenced by a family risk on top of reading fluency in grade 3. Because of the continuous nature of FR, we use reading scores of parents to quantify FR, in contrast to most previous studies which are based on distinct categories (high versus low FR). Finally, we explore whether grade 3 reading related factors, rapid automatized naming (RAN), phonological awareness (PA), and vocabulary, can further improve the prediction of grade 6 outcomes. As the importance of these reading related skills may vary during reading development, they may or may not explain extra variance in reading (e.g., de Groot, van den Bos, Minnaert, & van der Meulen, 2015; de Jong & van der Leij, 1999, 2003; Vaessen & Blomert, 2010, 2013). Below, we also discuss these predictors in more detail and formulate hypotheses about the role of these predictors.

## The continuity of reading ability and familial risk

Dyslexia is not a discrete disorder. There are multiple genes (e.g., Carrion-Castillo et al., 2017; Carrion-Castillo, Franke, & Fisher, 2013; Mascheretti et al., 2017) and multiple cognitive deficits that contribute to the disorder (e.g., Pennington, 2006; Pennington et al., 2012; van Bergen, van der Leij, & de Jong, 2014). Previous research has shown that the reading skills of people with dyslexia are part of the same continuum as reading skills of normal reading controls. In a population study, no bimodal distribution of reading skills could be found; instead, a normal distribution of reading skills was observed where dyslexia represented the lower tail of the distribution (Shaywitz, Escobar, Shaywitz, Fletcher, & Makuch, 1992b). The definition of dyslexia of the British Dyslexia Association also includes that dyslexia “is best thought of as a continuum, not a distinct category, and there are no clear cut-off points” (Rose, 2009, p. 30).

Since dyslexia is not discrete, FR is also continuous, as reading ability varies among parents, even within a group of parents with dyslexia. Several studies with categorical approaches to FR and reading ability have found poorer reading skills in a group of parents of children with a high FR who did develop dyslexia, compared to a group of parents of children with a high FR who did not develop dyslexia (Torppa, Eklund, van Bergen, & Lyytinen, 2011; van Bergen et al., 2011, 2012). This suggests that high FR children with dyslexia have a higher genetic liability for reading difficulties than high FR children without dyslexia.

Further evidence for the continuity of FR comes from studies that have demonstrated that some of the deficits found in high FR children with dyslexia are also found, although usually in a milder form, in high FR children without dyslexia, when compared to a low FR control group without dyslexia (e.g., Elbro, Borstrøm, & Petersen, 1998; Pennington & Lefly, 2001; Snowling, Gallagher, & Frith, 2003; van Bergen et al., 2012). For example, Snowling et al. (2003) found that high FR children without dyslexia performed significantly poorer on spelling, non-word reading, and reading comprehension compared to the low FR control group, although the difference between the high FR children with dyslexia and the low FR control group was larger. van Bergen et al. (2012) also found this stepwise pattern for spelling and word and pseudoword reading accuracy and fluency in grade 2 in children of the DDP. If FR was discrete, differences between the high and low FR groups without dyslexia would not be expected (see for a further discussion of this issue van Bergen et al., 2014).

In older advanced readers, who have received several years of reading instruction, differences between high FR and low FR groups without dyslexia are not always found. Whereas Snowling, Muter, and Carroll (2007) did find that high FR children without dyslexia at the age of 12 to 13 performed poorer than a low FR group without dyslexia on most literacy measures, including exception word reading, text reading accuracy, and timed measures of word and pseudoword reading fluency, Dandache, Wouters, and Ghesquière (2014) found only a significant effect for word reading fluency. Eklund, Torppa, Aro, Leppänen, and Lyytinen (2014) found no significant differences in reading and spelling between non-dyslexic high and low FR groups, although the raw scores of the high FR group were lower and effects sizes were small to moderate. As could be expected, in all studies, the two groups without dyslexia scored better than the high FR group with dyslexia. Thus, while some studies find small differences between high and low FR groups without dyslexia in advanced readers, these differences seem to be more pronounced among early readers. It has to be noted, however, that the studies cited above focus on different languages, English, Dutch, and Finnish, respectively, use different measures, contain different sample sizes, and use different criteria to diagnose dyslexia. The latter may be a crucial explanation for the different findings, because the stricter the criteria for dyslexia are, the more poor readers will be included in the high FR group without dyslexia. By using continuous measures of both FR and grade 3 reading fluency, we aim to avoid this problem in the present study.

## The predictive value of familial risk and grade 3 reading fluency

FR is the earliest risk factor that can be determined. Differences in early language development and pre-reading literacy related skills have been found among children with a familial risk of dyslexia who later developed dyslexia (see for a meta-analysis and review Snowling & Melby-Lervåg, 2016). Furthermore, some smaller differences in phonological skills, letter knowledge, vocabulary, and grammar have also been observed in pre-reading children with a high FR who later did not develop dyslexia and controls (Snowling & Melby-Lervåg, 2016). The question is how long FR contributes to prognosis and prediction in the personal history of a child, over and above the behavioral data on tests for reading and reading related cognitive skills. Because of the higher prevalence of reading problems among children with a high FR, and significant parent-child correlations in the studies by van Bergen et al. (2011) and Torppa et al. (2011), we expect that our continuous measure of FR is a significant predictor by itself for grade 6 reading outcomes (*Hypothesis 1*). Early reading problems tend to be persistent and are highly predictive for future reading outcomes (e.g., Ferrer et al., 2015; Shaywitz et al., 1999). For example, in a Belgian study with high and low FR children, strong correlations were found between grades 1, 3, and 6 for Dutch word and pseudoword reading fluency scores and moderate to high correlations across grades for spelling (Dandache et al., 2014). Therefore, we expect strong correlations between grade 3 reading fluency and grade 6 reading fluency (*Hypothesis 2*). An important next question is whether after introducing grade 3 reading fluency unexplained variance is left for FR to contribute to the prediction of grade 6 reading fluency independently. Because of the strong persistence of reading problems and because the previous studies discussed above have found only small, if any, effects of FR in adolescence, we expect that FR does not explain any variance in the grade 6 reading outcomes once we have controlled for grade 3 reading fluency (*Hypothesis 3*).

While dyslexia is defined as a reading and spelling problem at the word level, it may have consequences for reading comprehension. As the words in a text first need to be decoded before comprehension can take place, word decoding ability is considered to be an important predictor for reading comprehension according to the Simple View of Reading (Gough & Tunmer, 1986; Hoover & Gough, 1990), together with linguistic comprehension. Furthermore, the genetic variation in word decoding ability explains a large portion of the genetic variation in reading comprehension (Keenan, Betjemann, Wadsworth, DeFries, & Olson, 2006). A pathway analysis has also shown FR can influence reading comprehension via word reading fluency (van Viersen et al., 2018). This may explain why reading comprehension problems are also common among children with dyslexia, of whom some have a familial risk (e.g., Ferrer et al., 2015; Shaywitz et al., 1999). The analysis of longitudinal data has suggested that the relationship between reading fluency and reading comprehension is bidirectional (Klauda & Guthrie, 2008). The contribution of word decoding to reading comprehension generally decreases with age, when decoding becomes more automatic (García & Cain, 2014; Verhoeven & van Leeuwe, 2008). In the meta-analysis by García and Cain (2014), it was found that the average correlation between word decoding and reading comprehension in English was .74 and that age could explain 25% of the variance in the effect sizes. Based on these studies, we expect that grade 3 reading fluency also contributes significantly to the prediction of reading comprehension; however, in contrast to the effect for reading fluency, we expect the effect to be moderate as word reading is only one component of reading comprehension (*Hypothesis 4*).

## Rapid automatized naming, phonological awareness, and vocabulary

One of the most important predictors for reading fluency at the word level in advanced readers is RAN (van den Bos, Zijlstra, & Lutje Spelberg, 2002). A deficit in RAN, also referred to as naming speed, is frequently found among young children with dyslexia (e.g., Kirby, Parrila, & Pfeiffer, 2003; Landerl et al., 2013; Wolf & Bowers, 1999) and remains present in adulthood (e.g., Callens, Tops, & Brysbaert, 2012; van Setten, Martinez-Ferreiro, Maurits, & Maassen, 2016). RAN is also an important longitudinal predictor for reading in grade 6 (de Jong & van der Leij, 2003). Similarly, Dandache et al. (2014) found that RAN was poorer among children with dyslexia in grade 6 and, furthermore, that RAN could also predict the growth rate of the reading scores. Why RAN predicts reading skills is still under debate, although it is known that it measures more than just processing speed and articulation speed and is only moderately correlated with phonological skills (Norton & Wolf, 2012). Norton and Wolf (2012, p. 448) have suggested that it is “a microcosm of the reading system, providing an index of one’s ability to integrate multiple neural processes.” The importance of naming speed as a predictor for reading fluency in Dutch increases with age (Vaessen & Blomert, 2010, 2013). Furthermore, it is also predictive for reading among advanced readers with good reading skills (de Groot et al., 2015). In the present study, we only included a measure of alphanumeric RAN, naming digits, because especially alphanumeric RAN has been linked to literacy (e.g., Bowey, McGuigan, & Ruschena, 2005; Donker, Kroesbergen, Slot, van Viersen, & de Bree, 2016). The question that we will address in this paper is if RAN can explain variance in grade 6 reading fluency on top of grade 3 reading fluency and familial risk. Since we expect that especially grade 3 reading fluency will explain a lot of variance in grade 6 reading fluency but that the importance of RAN increases, we hypothesize that RAN will explain a small amount of extra unique variance in reading fluency (*Hypothesis 5*).

A deficit in PA is also frequently reported among people with dyslexia (e.g., Landerl et al., 2013; Ramus et al., 2003; Shaywitz & Shaywitz, 2005). PA has also been found to be the most discriminating factor between English-speaking adolescents with and without dyslexia (Shaywitz et al., 1999). For Dutch, Dandache et al. (2014) found that the phonological skills of high FR participants with dyslexia were significantly lower than those of both high FR and low FR participants without reading problems and that PA could also explain part of the growth in the reading skills between grade 3 and 6. On the other hand, de Jong and van der Leij (1999, 2002) showed that the influence of phonological abilities on word reading development is limited after grade 1, and they argue that PA is mainly related to the acquisition of accurate word decoding but not so much to the further development of word reading speed and fluency. Vaessen and Blomert (2010, 2013) have also found that while the importance of RAN for reading fluency increases with age, the importance of PA decreases. Similarly, a limited effect of PA on reading development after grade 1 has been found in other transparent languages like German (Landerl & Wimmer, 2008) and Norwegian (Lervåg, Bråten, & Hulme, 2009). Because PA has been found to be related to word reading ability, we expect that grade 3 PA is still correlated with grade 6 reading fluency and perhaps also (indirectly) with reading comprehension. However, because of the decreasing predictive value of PA for word reading fluency development found in most reviewed studies about transparent languages, and because of the overlap between PA and other variables, we expect that PA (in combination with RAN and vocabulary) is not a significant predictor for grade 6 reading fluency, once we have controlled for grade 3 reading fluency (*Hypothesis 6*).

Another factor that can contribute to the prediction of both word reading and reading comprehension is vocabulary knowledge. Nation and Snowling (2004) showed that besides phonological skills, oral language skills, including vocabulary, were both concurrent and longitudinal predictors for word reading and reading comprehension. This relationship may be reciprocal, as reduced reading experience, as a result of dyslexia, may lead to lower vocabulary knowledge. For example, Snowling et al. (2007) found that advanced readers with dyslexia had both lower vocabulary knowledge and lower print exposure than those without dyslexia. The importance of oral language skills for reading seems to increase with age, since language skills did not explain any variance on top of PA in grade 1, while they did in grade 6 (Ouellette & Beers, 2009). In transparent languages, linguistic comprehension is even for beginning readers a stronger predictor for reading comprehension compared to decoding accuracy but not stronger than decoding fluency (Florit & Cain, 2011). De Jong and van der Leij (2002) also found that vocabulary and listening comprehension skills were important for the development of reading comprehension after grade 1. Structural modeling has shown that vocabulary can contribute to reading comprehension, both by contributing to linguistic comprehension, and through its influence on word decoding (Tunmer & Chapman, 2012). According to the Lexical Quality Hypothesis (LQH), knowledge of word meaning is required for reading comprehension (Perfetti & Hart, 2001; Perfetti, 2007). In line with this hypothesis, Verhoeven and van Leeuwe (2008) found in a large-scale Dutch study that when word decoding, listening comprehension, and vocabulary skills were combined in one model, reading comprehension was predicted by all of them only in grade 1. In later grades, there was a reciprocal relationship between vocabulary and listening comprehension, and only vocabulary predicted reading comprehension directly. Based on these studies, we expect that vocabulary is moderately correlated with grade 6 reading comprehension and reading fluency. Because of the increasing importance of vocabulary, we also expect that vocabulary will be a significant predictor for reading fluency and reading comprehension in grade 6 once we have controlled for grade 3 reading fluency and the other reading related factors (*Hypothesis 7*).

In summary, in this longitudinal study, with a large sample of children with a high FR, we investigate how grade 6 reading fluency and reading comprehension can be predicted on the basis of a continuous measure of FR, grade 3 reading fluency, RAN, PA, and vocabulary. The above-formulated hypotheses are tested using a combination of correlation and regression analyses.

## Methods

### Participants

Of the 217 DDP participants who satisfied our inclusion criteria and completed the grade 3 measurements, 150 also participated in the grade 6 measurements and were included in the present study. The mean age in grade 3 was 8;11 (years; months), SD = 5 months, and in grade 6, it was 12;1, SD = 5 months. Sixty-seven participants were female and 83 were male. Although we do not use a categorical approach for the main analyses in this paper, children were divided into high and low FR groups at the start of the DDP, based on parental word and pseudoword reading fluency tests discussed further below. Parents were recruited through midwife offices during pregnancy, and parents were tested for dyslexia before their child was born. Just to illustrate that there is an overrepresentation of children with a high FR in our sample, compared to the normal population, we also report the number of participants per group and the criteria used. If parents reported that they had dyslexia as well as a family history of dyslexia, and if the reading fluency scores of a parent belonged to the lowest 20% on one test, and to the lowest 40% on the other test, using the decile norms by Kuijpers et al. (2003), a child was included in the high FR group (*n* = 83; 45 male). The parents of the children in the low FR control group (*n* = 67; 38 male) did not report a family history of dyslexia, and neither of the parents was diagnosed with dyslexia based on their reading fluency scores.

Children with comorbid developmental disorders such as attention deficit hyperactivity disorder (ADHD), attention deficit disorder (ADD), or autism spectrum disorders (ASD) were not excluded from the study, as comorbidity in dyslexia is common. Instead, we used the presence of comorbidity as a control variable in the analyses. In our sample, 21 children (eight low FR, 13 high FR) had one or more comorbid disorders, five had ASD, eight had ADD, nine had ADHD, and one had oppositional defiant disorder. Children with an IQ below 80 as measured during earlier DDP measurements were excluded from the study. Two children with severe medical or psychiatric conditions, as reported by the parents, were excluded (one in each FR group). One child with a high FR who did not cooperate during the measurements was also excluded.

The study was approved by the medical ethical review board of the University Medical Center Groningen. Parents gave informed consent for the participation of their children in this study; children who were more than 12 years old gave informed assent as well. Travel costs were reimbursed and children received a small toy or gift card to thank them for their cooperation.

### Materials

#### Reading fluency and familial risk

Both grade 3 and grade 6 reading fluency scores were measured with the same word and pseudoword reading tests. Word reading fluency was measured with a test consisting of a list of words that increased in length and difficulty (Brus & Voeten, 1973). The child had to read aloud as many words as possible correctly within 1 min. Pseudoword reading fluency was measured with a similar test, containing pronounceable non-existing words, also of increasing length and difficulty (“Klepel”; van den Bos, Lutje Spelberg, Scheepstra, & de Vries, 1994). In this case, as many pseudowords as possible had to be read aloud correctly within 2 min. For both tests, standardized norm-referenced Wechsler scores were obtained. A parallel-form reliability of .95 in grade 3 and .91 in grade 6 was found for pseudoword reading and .90 in grade 3 and .76 in grade 6 for word reading (van den Bos et al., 1994).

To quantify FR, the same word and pseudoword reading fluency tests were completed by parents. The mean decile norm score of the parent with the lowest reading scores was used. For some children in the high FR group, only the scores of the parent with dyslexia were present; in these cases, we assumed that this were the scores of the parent with the lowest reading ability. For children in the low FR group, it was required that scores of both parents were available such that we could ensure that there were no undiagnosed cases of dyslexia and could correctly identify the parent with the lowest reading scores.

The average reading scores of the parent with the lowest reading scores were negated by multiplying the scores with minus 1 to make the interpretation more intuitive. Thus, a higher FR score suggests a higher liability for reading problems.

#### Grade 3 reading related skills

In grade 3, several reading related skills were measured. PA was measured with a phoneme deletion task (de Jong & van der Leij, 2003). The children heard 27 pseudowords and had to delete one or two phonemes to make another pseudoword (for example, “memslos” minus “l” is “memsos,” and “urpgaap” minus “p” is “urgaa”). The score is the number of items reported correctly. A Cronbach’s alpha reliability of .80 has been found for children in grade 6 (P. de Jong, personal communication, February 5, 2018). RAN was tested with a serial digit naming task (van den Bos & Lutje Spelberg, 2007). The child received a card with 50 digits that had to be named as fast as possible. The number of errors and the time needed to complete the task were used to calculate the number of items correct per minute. A split-half reliability of .93 and a test retest reliability of .94 have been reported for this RAN task (van den Bos & Lutje Spelberg, 2007). Vocabulary was measured using the vocabulary task of the Dutch version of the Wechsler Intelligence Scale for Children, third edition (WISC-III-NL; Wechsler, 2005). Children were given a word and had to describe the word’s meaning. Standardized norm-referenced scores on a Wechsler scale were obtained. For this test, a Cronbach’s alpha of .80 and a split-half reliability of .88 have been reported for 8.5-year-old children, and the test-retest reliability has been found to be .77 for children between 6 and 8 years old and .85 for children between 9 and 11 years old (Wechsler, 2005).

#### Grade 6 reading comprehension

Reading comprehension was measured with a computerized test that the children performed at home. To this end, we used “Dia-tekst” (Dia-text), which is part of “Dia-taal” (Dia-language), an internet-based language testing package (Hacquebord, Stellingwerf, Linthorst, & Andringa, 2005). This test was completed by 130 of the 150 participants. There was no time limit for this test and the test could be interrupted and resumed later, such that the testing could be spread over multiple days and children could take a break whenever they wished. Parents were instructed that their child should complete the tests by themselves in a quiet environment. The test consisted of five short informative texts about various topics. There were 53 multiple choice questions that covered different aspects of the text; there were questions about propositions at the micro-level covering the comprehension of grammatical constructions and word level comprehension, questions about meso-level propositions covering the comprehension of relationships between sentences and small text fragments, and at the macro-level questions measuring the comprehension of the whole text. There were always three propositions from which the child had to choose the one that matched with (part of) the text. An overall national norm-referenced score was available for this test. The norms were based on the grade a child was in. A child is expected to gain 10 points per grade, so the mean score at the beginning of grade 6 is 51 and by the end of grade 6 it is 60 points. A Cronbach’s alpha reliability of .866 has been found for this test in a sample of 1053 children in grade 6 (H. Hacquebord, personal communication, March 2, 2017).

#### Background questionnaires

Background questionnaires were used to obtain information about the parental education level and the presence of comorbid disorders, which we controlled for in the regression. Information about the parental education level was obtained when parents signed up for the study. The parental level of education was measured on a scale from 0 to 4, ranging from only primary school to university level education. If this information was available for both parents, which was most often the case, we used the average level of education. Otherwise, we used only one score, and in one case where this information was missing completely, we replaced this value by the mean level of education in the regression analysis. A binary variable signifying the presence or absence of comorbid developmental disorders was created using information from a parental questionnaire during the grade 6 measurement. When there was no information available (*n* = 8), we assumed there were no comorbid disorders.

### Data analysis

First, we corrected all scores for age, since the norms we used were grade norms and not age norms, and since the children in the categorical high FR group were slightly older than the children in the low FR group in both grades (grade 3: *t* (148) = − 3.257, *p* = .001; grade 6: *t* (145.516) = − 4.107, *p* < .001), which was probably a result of repeating a grade. To this end, we used regression analyses with age in months as a predictor for all grade 3 and 6 test scores. The standardized residuals were saved and used as scores in all further analyses. Secondly, we created the grade 3 and grade 6 reading fluency score for each participant by averaging the word and pseudoword reading fluency residuals. We created one measure because these tests are highly correlated (grade 3: *r* = .848, *p* < .001, grade 6: *r =* .832, *p* < .001). Using two tests gives us a more reliable estimate of reading fluency as the amount of random error is reduced. Furthermore, word and pseudoword reading fluency could not be used as separate predictors because of multicollinearity.

To investigate the bivariate relationships between the different variables, we first performed a correlation analysis. To investigate how grade 6 reading fluency and reading comprehension can be predicted by a combination of variables, we performed hierarchical regression analyses. In the first step, the control variables, gender, parental education, and the presence of comorbid disorders were entered in the analyses. In the second step, the effect of FR was entered (*Hypothesis 1*) and in the third step the effect of grade 3 reading fluency was entered to investigate the effect of FR before and after the introduction of grade 3 reading fluency and to investigate the persistence and influence of reading problems on grade 6 outcomes (*Hypothesis 2–4*). In the fourth step, the grade 3 reading related skills, RAN, PA, and vocabulary were entered into the model to investigate whether they could contribute to the predictions of reading outcomes on top of grade 3 reading fluency and FR (*Hypothesis 5–7*). We entered these variables together since we did not have a specific hypothesis or research question that demanded that a certain variable should be entered first. It was investigated if interactions between FR and the other predictors improved the models, to study if the effect of these predictors differed depending on FR. Finally, using backwards regression, we investigated which combination of variables could explain the grade 6 outcomes best when non-significant predictors (*p* > .05) were removed one by one from the full model obtained in step 4 of the hierarchical regression analyses.

## Results

### Descriptive statistics and missing data analysis

In Table 1, descriptive statistics of the unstandardized variables can be found, such that the distribution of the variables can be inspected. Grade 6 reading fluency, composed of the grade 6 word and pseudoword reading measures, was normally distributed according to the Kolmogorov-Smirnov test, *D*(150) = .40, *p* > .200, but the distribution of grade 6 reading comprehension was not, *D*(130) = 2.12, *p* < .001, as it had a negative skew. For grade 6 reading fluency, there was one participant with an extremely high score of 19, and for grade 6 reading comprehension, there were 14 other cases with extreme scores in the lower tail. Of the predictors, only grade 3 RAN was normally distributed according to the Kolmogorov-Smirnov test, *D*(150) = .061. For vocabulary, there were two extreme outliers in the lower tail. Outliers were not removed since there were no theoretical reasons to do so, and these participants were considered part of the population of interest. Little’s MCAR test was used to investigate whether the 150 children who participated in grade 6 were different from the 67 children who did not participate in grade 6. For this test, we used all measures included in the regression analyses for the 217 participants who completed the grade 3 measurement. We found that grade 6 data were missing completely at random, *χ*^2^ (37) = 41.952, *p* = .265. An independent t-test also revealed that there was no significant difference in grade 3 reading fluency between participants who did participate in grade 6, and those who only participated in grade 3, *t*(215) = .229, *p =* .819*.* Also, when we only considered the data of the 150 participants who participated in grade 6, we found that the missing data (*n* = 20) on the computerized reading comprehension test was missing completely at random, *χ*^2^ (4) = 5.746, *p* = .219.

**Table 1:** Descriptive statistics of the unstandardized data

Variable	*N*	Min.	Max.	*M*	SD	Skewness (SE)	Kurtosis (SE)
Parental education	149	1	4	3.09	1.00	− .62 (.20)	− .98 (.39)
Familial risk	150	− 10	− 1	− 3.87	3.05	− .62 (.20)	− 1.19 (.39)
Grade 3 WRF	150	1	19	8.75	4.08	− .26 (.20)	− .50 (.39)
Grade 3 PWRF	150	1	16	8.47	3.46	− .08 (.20)	− .38 (.39)
Grade 3 PA	150	2	27	17.23	5.32	− .30 (.20)	− .42 (.39)
Grade 3 RAN	150	35	176	106.18	24.22	− .02(.20)	− .17(.39)
Grade 3 Vocabulary	150	4	17	12.00	2.40	− .22 (.20)	.23 (.39)
Grade 6 WRF	150	1	19	8.05	3.82	− .01 (.20)	− .33 (.39)
Grade 6 PWRF	150	1	19	9.13	3.60	.14 (.20)	− .19 (.39)
Grade 6 RC	130	32	78	66.18	1.82	− 1.68 (.21)	2.18 (.42)

*WRF* word reading fluency (Wechsler scale), *PWRF* pseudoword reading fluency (Wechsler scale), *RC* reading comprehension (norm-referenced score based on months of education), *PA* phonemic awareness (accuracy, max = 27), *RAN* rapid automatized naming (items correct/min)

Vocabulary is measured on a Wechsler scale. Familial risk is the average of the decile norm scores of parental WRF and PWRF multiplied with minus 1 such that a high score implies a high familial risk. Parental education is measured on a scale from 0 to 4

### Correlations

**Fig. 1:**
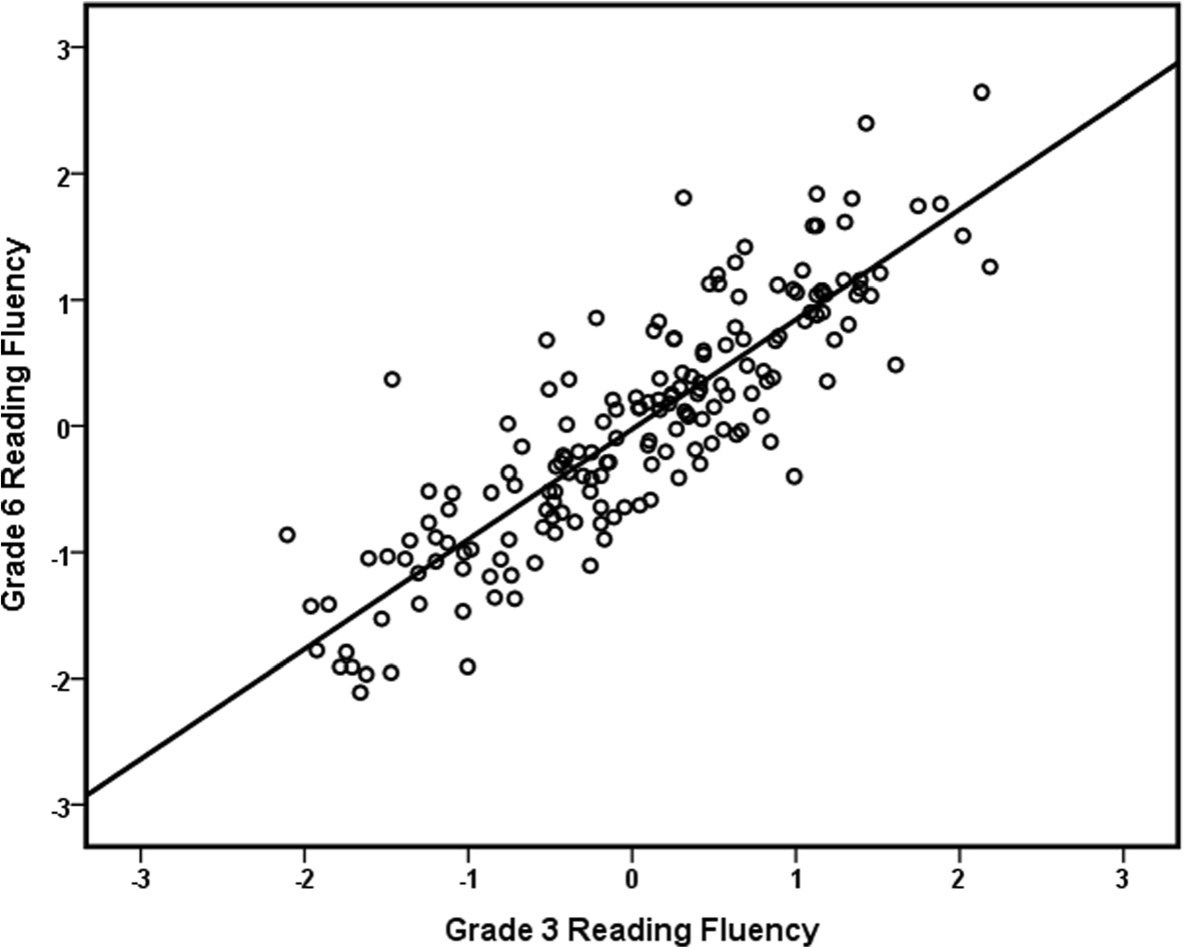
Scatterplot of the relationship between grade 3 reading fluency and grade 6 reading fluency. Scores on both axes are *Z* scores

**Fig. 2:**
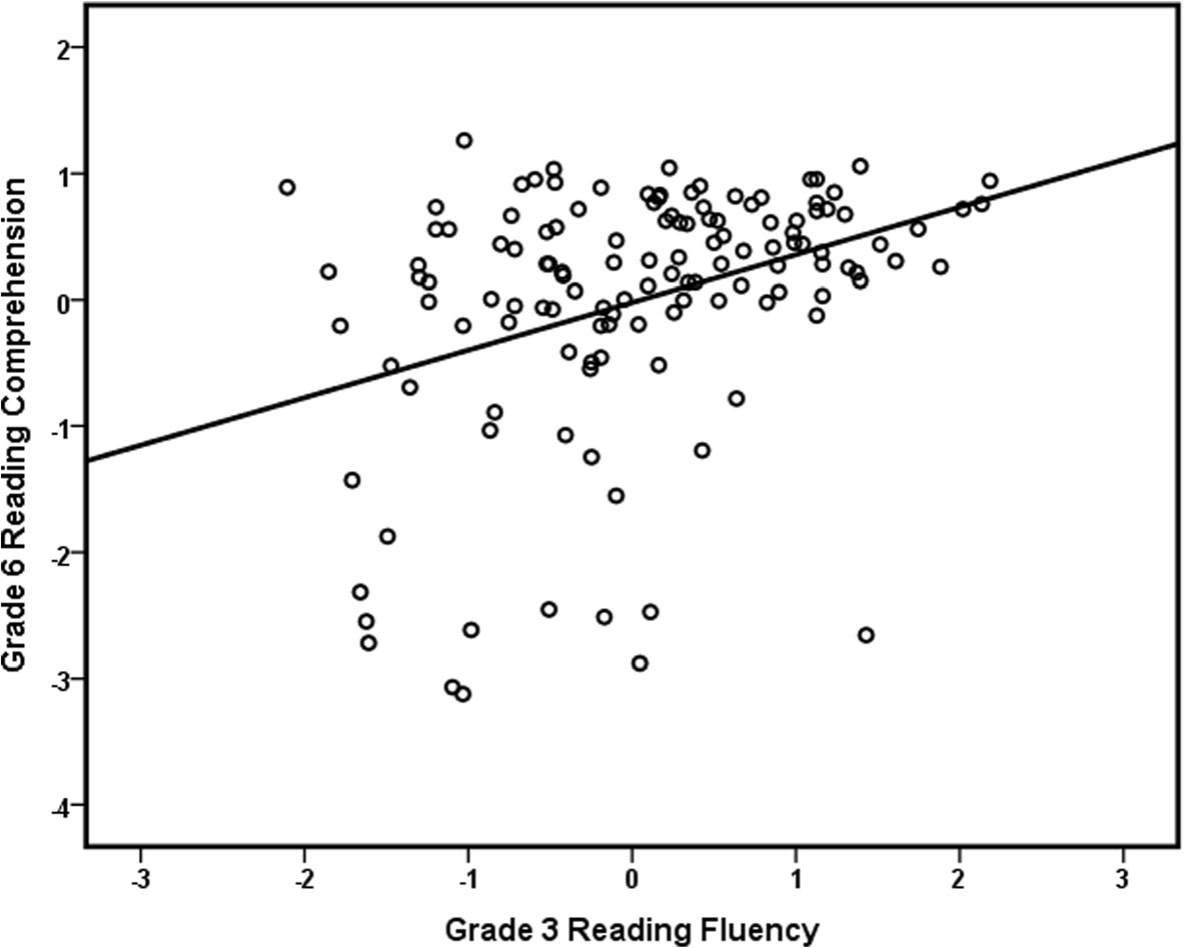
Scatterplot of the relationship between grade 3 reading fluency and grade 6 reading comprehension. Scores on both axes are *Z* scores

In Table 2, the correlations between control variables, grade 3 predictors, and grade 6 outcomes are displayed. The grade 6 reading fluency outcomes were significantly positively correlated with all grade 3 predictors (reading fluency, PA, RAN, and vocabulary), as predicted by *Hypotheses 2*, *5*, *6*, and *7*, and with the parental education level, but not with the presence of comorbid disorders and gender. Grade 6 reading comprehension was also significantly positively correlated with the same variables, except for grade 3 RAN, in line with *Hypotheses 4*, *6*, and *7*. Correlations between grade 6 reading fluency and comprehension outcomes and FR were significant and negative; a higher FR was associated with lower grade 6 outcomes, in line with *Hypothesis 1*. Grade 3 predictors were significantly positively correlated to each other. Scatterplots of the relationship between grade 3 reading fluency and grade 6 reading fluency and reading comprehension can be found in Figs. 1 and 2. Figure 1 shows that there is some variation at the individual level in reading fluency over the years, but overall, there is stability and there were no extreme outliers. Figure 2 shows that there is much more variation in grade 6 reading comprehensions in relation to grade 3 reading fluency and that there are a few children with poor comprehension skills despite reasonable reading fluency scores.

**Table 2:** Pearson correlation coefficients between grade 3 and grade 6 measures

	1	2	3	4	5	6	7	8	9	10
1. Gender	–									
2. Parental education	− .075	–								
3. Comorbid disorders	.002	− .134	–							
4. Familial risk	.126	− .514***	.052	–						
5. G3 RF	− .010	.274***	− .125	− .440***	–					
6. G3 PA	.042	.192*	− .158	− .290***	.578***	–				
7. G3 RAN	.031	.023	− .032	− .190*	.516***	.188*	–			
8. G3 vocabulary	− .087	.177*	− .232**	− .105	.305***	.272***	.186*	–		
10. G6 RF	.008	.227**	− .147	− .340**	.873**	.433**	.605**	.286**	–	
11. G6 RC	− .034	.197*	− .140	− .251**	.360***	.369***	.142	.366***	.336***	–

For all grade 3 and 6 test scores, the effect of age was removed by using the standardized residuals of a regression analysis with age in month as predictor

*G3* grade 3, *G6* grade 6, *RF* reading fluency, *PA* phonemic awareness, *RAN* rapid automatized naming, *RC* reading comprehension

*p < .05, **p < .01. ***p < .001

### Hierarchical regression analyses

In the first step of the hierarchical regression analyses, the control variables gender, parental education level, and the presence of comorbid disorders were entered in the model, as can be seen in the model summaries in Table 3. Only parental education level was a significant predictor for the grade 6 reading outcomes, as can be derived from the standardized regression coefficients in Table 4. The model with control variables was an improvement compared to an empty model only for reading fluency, but the amount of explained variance, 6.6%, was still quite small.

**Table 3:** Model summaries for the hierarchical regression models

Dependent variable	Model	*R*	*R* square	Std. error of estimate	*R* square change	*F* change	*df*1	*df*2	Sig. *F* change
G6 reading fluency	1	.257	.066	.933	.066	3.441	3	146	.019
	2	.371	.137	.899	.071	11.996	1	145	.001
	3	.876	.767	.469	.629	388.625	1	144	.000
	4	.897	.804	.435	.037	8.969	3	141	.000
G6 reading comprehension	1	.230	.053	.980	.053	2.349	3	126	.076
	2	.295	.087	.967	.034	4.648	1	125	.033
	3	.394	.155	.934	.068	10.023	1	124	.002
	4	.480	.231	.902	.076	3.968	3	121	.010

*G6* grade 6

Model 1: (constant) + gender + parental education level + presence of comorbid disorders. Model 2: model 1 + familial risk of dyslexia. Model 3: model 2 + grade 3 reading fluency. Model 4: model 3 + grade 3 rapid automatized naming + grade 3 phonological awareness + grade 3 vocabulary

**Table 4:** Standardized regression weights with significance and confidence intervals for the hierarchical regression models

		G6 reading fluency					Reading comprehension				
				95% confidence interval for *β*					95% confidence interval for *β*		
Model	Predictor	*β*	Sig.	Lower bound	Upper bound	sr^2^	*β*	Sig.	Lower bound	Upper bound	sr^2^
1	Gender	.024	.769	− .26	.351	.001	− .016	.854	− .377	.313	.000
	PE	.213	.009	.05	.356	.044	.182	.039	.009	.344	.033
	CD	− .119	.143	− .764	.111	.014	− .118	.178	− .879	.165	.014
2	Gender	.051	.511	− .197	.394	.003	.004	.962	− .334	.350	.000
	PE	.055	.541	− .118	.223	.002	.081	.409	− .109	.266	.005
	CD	− .124	.113	− .763	.082	.015	− .129	.139	− .904	.127	.016
	FR	− .312	.001	− .153	− .042	.071	− .211	.033	− .135	− .006	.034
3	Gender	.01	.814	− .136	.173	.000	− .020	.814	− .371	.292	.000
	PE	.007	.878	− .082	.096	.000	.073	.440	− .110	.253	.004
	CD	− .038	.358	− .325	.118	.001	− .085	.318	− .761	.249	.007
	FR	.056	.269	− .014	.049	.002	− .092	.369	− .098	.037	.006
	G3 RF	.891	.000	.815	.997	.629	.292	.002	.115	.498	.068
4	Gender	.009	.813	− .127	.162	.000	− .020	.808	− .368	.287	.000
	PE	.034	.442	− .051	.116	.001	.030	.752	− .150	.208	.001
	CD	− .047	.226	− .339	.081	.002	− .017	.839	− .554	.451	.000
	FR	.056	.236	− .012	.047	.002	− .097	.329	− .098	.033	.006
	G3 RF	.823	.000	.722	.951	.292	.128	.288	− .115	.383	.007
	G3 RAN	.202	.000	.105	.267	.029	− .033	.730	− .213	.150	.001
	G3 vocabulary	.009	.825	− .069	.086	.000	.240	.009	.060	.405	.045
	G3 PA	− .082	.084	− .171	.011	.004	.185	.078	− .022	.402	.020

*G3* grade 3, *G6* grade 6, *PE* parental education level, *CD* presence of comorbid disorders, *FR* familial risk of dyslexia, *RF* reading fluency, *RAN* rapid automatized naming, *PA* phonological awareness, s*r*^*2*^ squared semi-partial correlation

In the second step, FR was entered into the model. This was a significant negative predictor and resulted in a significant improvement of the models for all grade 6 outcomes; this is also in accordance with *Hypothesis 1*. On top of the control variables, FR explained 7.1 and 3.4% of variance in reading fluency and, respectively. Interestingly, the effect of parental education level disappeared completely. This can be explained by the fact that parental education level and FR were moderately correlated, and both parental education level and FR were related to grade 6 reading fluency and reading comprehension. This means that there is no unique contribution of parental education level to the prediction of grade 6 reading outcomes if FR is taken into account, as can also be seen from the squared semi-partial correlations in Table 4.

In the third step, we entered grade 3 reading fluency to investigate how much extra variance can be explained by the previous reading level and to investigate if the relationship between FR and the reading outcomes changes when grade 3 reading fluency is introduced. As can be seen in Table 3, adding grade 3 reading fluency improves the model fit significantly for both reading outcomes. Like predicted by *Hypothesis 2*, the increase in explained variance for reading fluency was substantial, with 62.9%. The increase in explained variance in reading comprehension was only modest with 6.8%, like *Hypothesis 4* stated. Interestingly, the significant effect of FR that we found in step 2 for reading fluency and reading comprehension completely disappeared as a result of the introduction of grade 3 reading fluency, in accordance with *Hypothesis 3*, as can be seen in Table 4. Since FR is by itself significantly related to the grade 6 outcomes and grade 3 reading fluency, and grade 3 reading fluency is also significantly related to the grade 6 reading outcomes, it seems that there is no unique effect of FR once we control for grade 3 reading fluency. By looking at the squared semi-partial correlations in Table 4, it can be seen that reading fluency has a large unique contribution to the prediction of reading fluency and comprehension, while the unique effect of other predictors has completely disappeared.

In the fourth step of the analysis, the grade 3 reading related factors, RAN, PA, and vocabulary were entered, testing *Hypotheses 5*, *6*, and *7*. This resulted in a significant improvement of the model for all outcomes, as can be seen in Table 3. The explained variance increased with 3.7% for reading fluency and 7.6% for reading comprehension. Table 4 shows that PA was not a significant predictor for both reading fluency and reading comprehension, which is in line with *Hypothesis 6*, although the effect for reading comprehension approached significance (*p* = .078), and it had a small unique contribution to the prediction of reading comprehension based on the squared semi-partial correlation. RAN was found to be a significant positive predictor for reading fluency; better RAN scores are related to better word reading outcomes as stated in *Hypothesis 5*. However, grade 3 reading fluency was still a better predictor and had a larger unique contribution to the prediction of grade 6 reading fluency. It should be noted though that the unique contribution of grade 3 reading fluency in model 4 is much lower as the result of the overlap with the newly included predictors that we also found in the correlation analysis. For reading comprehension, the only significant positive predictor in the fourth model is grade 3 vocabulary, which is in accordance with *Hypothesis 7*. The significant effect of grade 3 reading fluency on reading comprehension in the previous steps now disappears. Furthermore, the squared semi-partial correlations show that it has no unique contribution to the prediction of reading fluency anymore. As grade 3 reading fluency and vocabulary are significantly related to each other and to reading comprehension, it seems that vocabulary can explain some of the same variance in reading comprehension as grade 3 reading fluency. However, when only vocabulary and grade 3 reading fluency are included as predictors for reading comprehension, in a separate analysis, the effect of grade 3 reading fluency is still significant. Thus, the explained variance of vocabulary and grade 3 reading fluency does not overlap completely, and the explained variance of reading fluency must be partially overlapping with at least some of the other non-significant predictors as well. However, the full amount of variance that grade 3 reading fluency can explain in reading comprehension can also be explained by vocabulary in combination with PA, which was not a significant predictor in this model, but was significant in the backwards model described below.

Finally, interactions between FR and the grade 3 predictors (grade 3 reading fluency, RAN, PA, vocabulary) were investigated. Since these interactions did not improve the model fits, these results are not further discussed.

### Backward regression analyses

Backward regression was used to obtain a model with only significant predictors for reading fluency and reading comprehension. Regression coefficients for these models can be found in Table 5. For reading fluency, this resulted in a model with grade 3 reading fluency and RAN, which explained 79.5% of the variance. While reading fluency explains a large portion of unique variance, RAN explains only a limited amount of unique variance in this model based on the squared semi-partial correlations. This can be explained by the fact that there is quite a strong correlation between these two predictors, as can be seen in Table 2. The model for reading comprehension contained vocabulary and PA as predictors and explained 20.4% of the variance. Note that grade 3 PA only became a significant predictor for reading comprehension after non-significant variables were removed. Both variables explained a similar amount of unique variance, although there was also some overlap since there is a small positive correlation between these variables, as can be seen in Table 2.

**Table 5:** Standardized regression coefficients, significance, and confidence intervals for the final models obtained using backward regression

G6 dependent variable	G3 predictor	*β*	Sig.	95% confidence interval for *β*		sr^2^
				Lower bound	Upper bound	
Reading fluency	Reading fluency	.765	.000	.690	.865	.429
	RAN	.211	.000	.114	.272	.032
Reading comprehension	Vocabulary	.275	.001	.106	.427	.068
	PA	.279	.001	.117	.457	.070

*G6* grade 6, *G3* grade 3, *RAN* rapid automatized naming, *PA* phonological awareness, *sr*^*2*^ squared semi-partial correlation

## Discussion

In the present study, we investigated how grade 6 reading outcomes, reading fluency (and reading comprehension, can be predicted on the basis of grade 3 reading fluency, FR, and grade 3 reading related measures: RAN, PA, and vocabulary. In contrast to previous studies that used a dichotomous distinction between children with or without dyslexia, we used continuous measures of reading fluency to quantify the children’s reading ability and we based FR on parental reading fluency scores. We found a strong persistence of reading problems at the word level, as grade 3 reading fluency was highly predictive for grade 6 reading fluency. Furthermore, higher reading comprehension scores in grade 6 were associated with a higher level of grade 3 reading fluency. With respect to the role of FR in the prediction of the grade 6 reading outcomes, we conclude that, although FR is by itself related to the grade 6 outcomes, FR did not explain any variance on top of grade 3 reading fluency, nor did the interaction between FR and grade 3 reading fluency. Of the grade 3 reading related measures, RAN was an extra predictor for reading fluency in grade 6, whereas vocabulary and PA were the main predictors for reading comprehension skills. Below we discuss the hypotheses that we formulated in the introduction in more detail.

### Familial risk and grade 3 reading fluency

Significant negative correlations between FR and the grade 6 reading outcomes were found, in accordance with *Hypothesis 1*. The correlation was moderate for reading fluency and small for reading comprehension. This result is in line with the higher prevalence of reading problems among children with a parent with dyslexia (Snowling & Melby-Lervåg, 2016). Noteworthy is that the parental education level was also related to the parental reading level; better reading parents, i.e., with a lower FR, had a higher level of education. This is probably a bidirectional relationship, as people with better reading skills obtain a higher level of education, but reading skills may also improve as the result of this education. Nevertheless, the effect of FR cannot be solely attributed to the parental education level since the effect of FR was significant in the hierarchical regression analysis where we controlled for parental education level in the previous step. In the present study, we operationalized the familial risk by using only parental reading scores. Yet, although there are many studies that used parental reading scores to measure familial risk, though usually categorically, it would be worthwhile to investigate if there are more parental factors that could be related to the children’s reading outcomes and perhaps also reading development. This could be measures of environmental factors, like aspects of SES other than parental education, for which we already controlled in this study or biological measures including gene expression and neurological factors. Also cognitive factors are of interest, because they may reveal underlying deficits at the cognitive level which are no longer present at the behavioral level because of compensation. For example, parental RAN also differed between children with a high FR with and without dyslexia in the study by van Bergen et al. (2012) and could be included in a future study as an additional risk factor.

The strong relationship between grade 3 reading fluency and grade 6 reading fluency indicates that reading ability is already quite stable in grade 3 and thus that reading problems tend to be persistent into early adolescence. This is in line with previous studies (e.g., Shaywitz et al., 1999; Snowling et al., 2007; Dandache et al., 2014; Eklund et al., 2014) and confirms *Hypothesis 2* that grade 3 reading fluency is a strong predictor for grade 6 reading fluency. We expected that FR would not explain any variance in the grade 6 reading outcomes once we controlled for grade 3 reading fluency (*Hypothesis 3*). This hypothesis was indeed confirmed, as FR did not predict any variance on top of grade 3 reading fluency for both grade 6 outcomes. Therefore, we conclude that the effect of FR was already captured in grade 3 reading fluency. However, this does not mean that FR becomes less important for reading in grade 6. FR is equally important for grade 3 and grade 6 reading, but once grade 3 reading fluency is known, FR does not explain any unique variance. This finding is in line with the study of Eklund et al. (2014) where the effect of risk was not significant either, as they did not find any significant differences between high and low FR advanced readers without dyslexia. However, in contrast to previous studies, we used continuous measures of reading ability and FR based on (parental) word and pseudoword reading fluency. Therefore, we can now rule out that our results are biased by an arbitrary distinction between dyslexia and normal reading ability. Thus, we were able to replicate previous findings, which are very valuable for the scientific process, and have demonstrated the usefulness of measuring reading ability and FR as continuous variables.

Children with a high grade 3 reading fluency were also more likely to score better on reading comprehension in grade 6. However, the correlation was moderate and not strong like it was for reading fluency. This finding confirms *Hypothesis 4* that grade 3 reading fluency can also moderately contribute to the prediction of reading comprehension. We expected that the variance explained by grade 3 reading fluency in grade 6 reading comprehension would be lower than in grade 6 reading fluency because word reading is only one of the processes needed for comprehension and because the importance of word reading for reading comprehension decreases with age (Verhoeven & van Leeuwe, 2008). In a further step in the hierarchical regression analysis, we found that grade 3 reading fluency did not explain unique variance in reading comprehension but that the variance explained by reading fluency could also be fully explained by vocabulary and PA. Nevertheless, reading comprehension problems are associated with word reading problems, which should not be ignored in clinical practice.

### Reading related skills

Despite the fact that a lot of variance at the word level was already explained by grade 3 reading fluency, RAN still contributed to the prediction of grade 6 reading fluency. This confirms *Hypothesis 5* that RAN would be related to these reading outcomes and that it would explain additional variance in reading fluency on top of grade 3 reading fluency. However, the amount of extra variance that was explained by RAN was limited, probably as a result of the strong stability in reading fluency. These results are in line with the findings by Vaessen and Blomert (2010, 2013) that the importance of RAN increases with age, the study by de Groot et al. (2015) which showed that RAN predicts reading fluency skills across the whole reading fluency continuum, and with the study by Dandache et al. (2014) where RAN significantly predicted the growth in reading development. The significance of RAN in this model means that there is unexplained variance in the grade 6 reading outcomes once we controlled for the grade 3 reading fluency level and other variables and that RAN can explain a small part of this variance. Therefore, there has to be an aspect of RAN that is only related to grade 6 reading fluency that was not yet related to reading fluency in grade 3, because otherwise the effect of RAN would have been captured already in grade 3 reading fluency. This interpretation seems to make sense if RAN reflects the automatization of reading. Alternatively, it could be a result of maturation which may increase processing speed both during reading and RAN. As we discussed, it is still under debate which reading related subskills are reflected in RAN exactly (Norton & Wolf, 2012). In a recent study (Papadopoulos, Spanoudis, & Georgiou, 2016), the relationship between RAN and reading fluency was investigated for grade 1, by looking at a combination of multiple possible underlying processes. Here, RAN had both direct and indirect effects on oral reading fluency, through phonological awareness and orthographic processing. However, for advanced readers, more systematic investigations into the relationship between RAN and reading fluency are still needed to understand why RAN is an important predictor for advanced reading skills. RAN was not significantly related to reading comprehension in our study, in accordance with the study by de Jong and van der Leij (2002).

With respect to PA, we hypothesized that PA would be correlated with grade 6 reading fluency and perhaps also (indirectly through word reading ability) with reading comprehension but that it would not play a role in the prediction of the grade 6 reading outcomes in a model with grade 3 reading fluency (*Hypothesis 6*). Indeed, we found a positive correlation between PA and grade 6 reading fluency, but PA did not explain unique variance in reading fluency on top of the other predictors, in line with previous studies (e.g., de Jong & van der Leij, 1999, 2003; Vaessen & Blomert, 2010, 2013). Interestingly, PA was not only correlated with reading comprehension; it was also a significant predictor for reading comprehension in the backward regression analysis. Here, PA was a better predictor than grade 3 reading fluency for grade 6 reading comprehension. PA and grade 3 reading fluency are significantly correlated, which can explain why the effect of PA became significant when grade 3 reading fluency was excluded. According to the LQH, rich phonological representations are part of a word’s lexical quality and a higher lexical quality has a positive influence on reading comprehension. Therefore, better PA skills, leading to richer lexical representations may also contribute to better reading comprehension.

We hypothesized that vocabulary would be related to both reading outcomes and that it would also be a significant predictor for reading fluency and reading comprehension (*Hypothesis 7*). We found that vocabulary was indeed significantly correlated to both outcomes; the correlation was moderate for reading comprehension, but unlike we hypothesized small but close to moderate for reading fluency. Vocabulary did also not further contribute to the prediction of grade 6 WRF. Some vocabulary related variance was probably captured in grade 3 reading fluency, since WRF was part of grade 3 reading fluency, and some of the remaining variance was already explained by RAN. Nevertheless, grade 3 vocabulary proved to be an important predictor for grade 6 reading comprehension. In fact, it was the only significant predictor in the full regression model, and as already discussed, the same variance in grade 6 reading comprehension that can be explained by grade 3 reading fluency could also be explained by grade 3 vocabulary, together with PA. The importance of vocabulary for comprehension is in line with the LQH (Perfetti & Hart, 2001; Perfetti, 2007) that states that rich lexical representations are needed for comprehension; vocabulary entails the semantic part of these representations. It should be noted, however, that we did not have a measure of the previous reading comprehension level, like we had for reading fluency. Therefore, we cannot determine if, and to what extent, growth in reading comprehension is explained by vocabulary. It is likely that vocabulary explained at least some variance that grade 3 reading comprehension would have explained, if it were included. Because growth in reading comprehension was not assessed, we cannot establish a causal effect of vocabulary on reading comprehension. However, we can say that vocabulary is an important indicator of grade 6 reading comprehension.

### Limitations and implications

There are a few drawbacks of this study. First, we did not have any control over the (quantity and quality of) the help that was given to the children with reading problems. Since interventions may have influenced the outcomes, this would be an interesting topic for further studies. Secondly, in the present study, we only looked at the reading scores of one parent because the reading scores of both parents were not always available for the children with a high risk. It would be interesting to consider the reading scores of the other parent as a separate predictor, as well. However, we expect that the amount of explained variance by the second parent’s reading scores is smaller than the amount of variance explained by the reading scores of the first parent, because previous research has found a small but significant correlation between the reading skills of parents (van Bergen, Bishop, van Zuijen, & de Jong, 2015). Thirdly, a drawback of the reading comprehension test that we used was that we could not control the environment and the circumstances under which the child made the test, because the children made the test at home because of time concerns. Parents were instructed that children should do the test by themselves, but it is impossible to verify whether this actually happened. On the other hand, we were able to use quite a long test that consists of multiple texts, such that the knowledge about a certain topic did not strongly influence the results. Also, because the distribution of the reading comprehension test was negatively skewed and leptokurtic, which suggests that there could be a ceiling effect, we should be cautious when interpreting the results with respect to reading comprehension. Therefore, we suggest using a different reading comprehension test in a replication study. In such a study, multiple measures per variable, like RAN and PA, could also be included to reduce the amount of measurement error.

A drawback of linear regression is that the regression coefficients are estimated for people with an average level of the dependent variable, while it could be that the relationships found in this study are different for people with low or high scores. This would be an interesting question to investigate further with quantile regression in a larger sample. In this respect, it has to be noted that children with a high FR were overrepresented in the present study. This is actually useful, since longitudinal data collection is a time-consuming process, and it increases the study’s power with respect to FR. Thus, we can be confident that the main result—the limited variance explained by FR once grade 3 reading fluency is accounted for—which was the main focus of the study is valid. However, the predictive value of RAN, PA, and vocabulary could be overestimated due to overrepresentation of FR children, in comparison with the general population. Since the interactions with familial risk did not improve the models for reading fluency and reading comprehension, we can conclude though that there are no large differences in the models for people with and without a familial risk. Still, a large-scale study in a normal population would be needed to confirm the generalizability of our results.

Future studies could also use structural equation modeling to improve the understanding of how both the dependent and independent variables are related to each other, because most measures were interrelated. Ideally, one would need a larger sample for these analyses. In hindsight, it would have been interesting to include other predictors as well, such as the visual attention span for reading fluency and grade 3 measures of reading and listening comprehension for grade 6 reading comprehension outcomes, which could be done in future studies. Finally, it would be interesting to investigate reading in advanced readers further in different orthographies and educational systems. Our results do support the results of earlier predictive Dutch studies (Dandache et al., 2014; de Jong & van der Leij, 1999, 2002) and of studies in other languages (e.g., Snowling et al., 2007; Landerl et al., 2013; Eklund et al., 2014). Because there are many methodological differences between these studies, a more systematic comparison is still needed.

There are several conclusions that can be drawn from this study about the development of grade 6 reading skills that have practical implications. First of all, based on our results, we expect that children with a low reading fluency level in grade 3 still have reading problems at the end of primary school in grade 6. Special arrangements for these children, such as extra time in test situations and the use of assistive technology, will thus often remain necessary. As we found that children with a high FR do not have a worse prognosis than children with a similar grade 3 reading fluency but a low FR, extra monitoring of these children with a high FR after grade 3 does not seem to be necessary. This is not to say that screening for reading problems after grade 3 should be completely abolished, as there may still be individual cases of late emerging reading problems, especially in reading comprehension. RAN did improve the prediction of reading fluency, but the amount of extra variance explained was limited, so it may be used to confirm reading problems and asses the underlying cognitive deficit, but it is not very useful for screening or monitoring purposes once earlier reading fluency scores are available. In the case of reading comprehension, vocabulary turned out to be the best predictor, which explained a substantial amount of variance, and it may therefore be especially interesting for screening purposes and, if a causal effect of vocabulary can be established, also for intervention purposes. Finally, we have shown that dyslexia and the risk for dyslexia can be treated as continuous constructs, which has resulted in an approach to dyslexia that more closely resembles how reading difficulties manifest themselves in real life and probably in more statistical power. We therefore believe that a continuous approach to dyslexia should be used more often, both in research and in clinical practice.
